# Cardiovascular adverse events associated with antibody-drug conjugates (ADCs): a pharmacovigilance study based on the FAERS database

**DOI:** 10.3389/fphar.2024.1378010

**Published:** 2024-05-03

**Authors:** PingPing Long, Siyu Li, Lingyun Pan, Yuanqiang Wang, Wanyi Chen, Xiaoxiao Wang

**Affiliations:** ^1^ School of Pharmacy and Bioengineering, Chongqing University of Technology, Chongqing, China; ^2^ Department of Pharmacy, Chongqing University Cancer Hospital, Chongqing, China

**Keywords:** FAERS, disproportionality analysis, antibody-drug conjugate, cardiovascular toxicity, adverse events

## Abstract

**Objective:**

As a novel drug formulation, antibody drug conjugates (ADCs) are widely used in various types of cancer. However, clinically, there is a lack of attention to the CVD produced by them, as well as a lack of research on the real-world situation. Using the Food and Drug Administration Adverse Event Reporting System (FAERS) database, to ensure its clinical safety application, we analyzed post-marketing data on antitumor ADCs to identify risk factors and drugs associated with the risk of cardiovascular events.

**Research design and methods:**

We used OpenVigil 2.1 to conduct a database query for adverse events (AEs) reported to the FAERS database between the time the drug was launched and the second quarter of 2023. Cardiovascular adverse events (AEs) were grouped into fourteen narrow categories using the Standardized Medical Dictionary for Regulatory Activities (MedDRA) Queries (SMQs), and the reporting odds ratio (ROR) and the proportional reporting ratio (PRR) for reporting the association between different drugs and cardiovascular disease (CVD) risk were calculated.

**Results:**

In the FAERS database, 1863 AEs associated with CVD we studied were identified in patients receiving ADC therapy. Most reports came from people aged ≥65, but a significant number of cases were found to be unknown. The number of patients with antibody-drug conjugates (ADCs)-related CVD cases aged <18 years, 18–64 years, and≥ 65 years was 52 (2.79%), 586 (31.45%), and 613 (32.90%), respectively. The proportion of female patients (834, 44.77%) was higher than that of male patients (752, 40.37%). Death (770 reports), disability (9 reports), Hospitalization initial or prolonged (407 reports), and life-threatening reactions (187 reports). Of the 770 deaths reported, 103 (31.7%) were associated with brentuximab vedotin, 10 (24.4%) with sacituzumab govitecan, 22 (19.3%) with enfortumab vedotin, and 35 (34.7%) with trastuzumab emtansine.49 (41.2%) cases were associated with polatuzumab vedotin, 62 (29%) with trastuzumab deruxtecan, 423 (54.3%) with gemtuzumab ozogamicin, and 66 (38.8%) with inotuzumab ozogamicin. In a disproportionate number of SMQS, cardiac failure (*n* = 277) and embolic and thrombotic events, venous (*n* = 446) were the most frequently reported CVD-related AEs in ADCs.

**Conclusion:**

By mining the FAERS database, we provided relevant information on the association between ADC use and cardiovascular-associated AEs. ADCs were associated with increased cardiovascular toxicity, deserving distinct monitoring and appropriate management. Further research is needed to confirm these findings and assess causality.

## 1 Introduction

Antibody drug conjugates (ADCs), as a new type of anti-cancer drugs, are widely used in various cancers, including hematologic neoplasms and solid tumors, and have been described as a “magic bullets” for cancer treatment (Chen et al., 2022; Johns and Campbell, 2022; Dumontet et al., 2023). ADC consists of a linker, payload, and monoclonal antibody (mAb) (Fu et al., 2022). It combines the advantages of high-specific targeting ability and strong killing effect, enabling precise and efficient elimination of cancer cells, and has become one of the hotspots in anticancer drug research and development (Fu et al., 2022). Due to the fact that, compared to traditional cytotoxic chemotherapy, ADCs enhance the cytotoxic effect and targeting ability, while reducing the toxicity of anti-tumor drugs, ADCs have been developed (Beck et al., 2017). However, although ADCs are a type of targeted chemotherapy, higher toxicity and adverse side effects have been reported (Tarantino et al., 2022). Adverse cardiovascular events will have a serious impact on the quality of life and long-term survival time of patients and may also cause serious and life-threatening outcomes (Markina et al., 2023), which will seriously affect the prognosis of tumor patients. Evidence from the FAERS database suggests that ADCs cause cardiovascular toxicity. A recent study showed that the cardiotoxicity of ADCs with a payload of Trastuzumab deruxtecan (T-DXd) was consistently reported (Soares et al., 2023). Although ADCs are commonly prescribed and widely used in clinical settings, the risk of their development into CVD remains controversial. In some studies, the correlation between Trastuzumab deruxtecan and Trastuzumab emtansine and cardiotoxicity has been reported; however, it has not yet been confirmed (Ma et al., 2023; Soares et al., 2023). In addition, the cardiotoxicity risk of other ADCs remains unclear.

Components of cardiovascular toxicity include cardiomyopathy and heart failure, myocarditis, arrhythmias, coronary artery disease, early-onset valvular disease, hypertension, and thromboembolism (Alexandre et al., 2020). In the past, there have been studies on the mining of ADCs adverse events based on the FAERS database, both at home and abroad. One study only mined the signal of adverse events for two ADCs; another study focused on liver injury from ADCs (Ma et al., 2023; Sun et al., 2023), but no study has yet analyzed cardiovascular adverse events related to ADCs using the FAERS database. Therefore, it is necessary for this study to attempt to analyze cardiovascular adverse events related to ADCs.

A comprehensive summary of ADCs based on real-world data is therefore necessary to identify safety risk signals, particularly adverse drug reactions (ADRs), that are not included in safety instructions or specifications. Limited information has been reported regarding the detection and evaluation of cardiovascular associations related to ADRs signals associated with ADCs. Currently, the FAERS database is the largest repository worldwide for spontaneously reported adverse events and is widely recognized for its extensive and standardized data. FAERS can be utilized for ADRs analysis, while data mining algorithms have been employed to conduct post-marketing safety monitoring and reevaluation of drugs from AEs databases. Hence, this pharmacovigilance analysis was designed to systematically investigate potential red flags indicating cardiovascular adverse events associated with ADCs. The objective of this study is to enhance the safe prescribing practices of ADCs by mining and evaluating relevant signals from the FAERS database, which contains anonymous patient information confirmed by hospital ethics committees as not requiring ethical approval.

## 2 Materials and methods

### 2.1 Data source

Following approval from the U.S. Food and Drug Administration (FDA) in 2000, the first ADC, mylotarg (gemtuzumab ozogamicin), was introduced. Since then, a total of 15 ADCs have been approved by the FDA for treating various types of cancer (Table 1). This retrospective study analyzed publicly available AEs data from the FAERS database from the time after each drug was launched to the second quarter of 2023 using OpenVigil 2.1. This tool, intended for healthcare professionals, enables visual access to FAERS pharmacovigilance data. OpenVigil 2.1 is a web-based query tool for physicians and pharmacists that visually accesses FAERS pharmacovigilance data. The pharmacovigilance tool OpenVigil2.1 enables researchers to extract, clean, mine, and analyze structured AEs information directly from the FAERS database through an API, transforming large raw FAERS datasets into a form that can be extracted and analyzed (Böhm et al., 2012). The FAERS database pharmacovigilance tool OpenVigil2.1, embedded with MedDRA version 24.0, also provides a variety of AEs retrieval methods, including standard MedDRA analytical queries (broad), standard MedDRA analytical queries (narrow), high, preferred, and low. All searches in this study were conducted using standard MedDRA analytical queries (narrow) in the OpenVigil2.1 tool. The operation is relatively simple, and the data is summarized every quarter. In this study, we searched for the gender of the patient, the age at the onset of AEs, the date the report was received, the country in which it was reported, the event, and the outcome. However, the diversity of patient ethnicities and regions may affect the study’s generalizability.

**TABLE 1:** The FDA has granted approval to 15 classes of Antibody-drug Conjugates.

Trade names	Common name	Company	Target antigens	Payloads	Linkers	Approved indications	Approved date
Mylotarg	gemtuzumab ozogamicin	Pfizer	CD33	N-acetyl-γ-calicheamicin	hydrazone	Newly diagnosed positive for CD33	2000.05 2017.09
Adcetris	brentuximab vedotin	Seattle	CD30	MMAE	dipeptide	hodgkin lymphoma (hl)	2011.08
Kadcyla	trastuzumab emtansine	Roche	HER2	DMI	thioether	HER2 positive breast cancer	2013.02
Besponsa	inotuzumab ozogamicin	pfizer	CD22	N-acetyl-γ-calicheamicin	hydrazone	r/rB cell acute lymphoblastic leukemia	2017.06
Lumoxiti	moxetumomab pasudotox	AstraZeneca	CD22	PE38	mc-VC-PABC	r/r hair-cell leukemia	2018.09
Polivy	polatuzumab vedotin	Roche	CD79B	MMAE	mc-VC-PABC	r/r diffuse large B-cell lymphoma	2019.06
Padcev	enfortumab vedotin	Seattle	Nectin-4	MMAE	Mc-VC-PABC	Advanced urothelial carcinoma	2019.12
Enhertu	Fam-trastuzumab deruxtecan	AstraZeneca	HER2	Dxd	tetrapeptide	Metastatic HER2-positive breast cancer	2019.12
Trodelvy	sacituzumab govitecan	Immunomdecis	TROP-2	SN38	CL2A	Triple negative breast cancer	2020.04
Blenrep	belantamab mafodotin	GSK	BCMA	MMAF	mc	r/r multiple bone marrow cancer	2020.08
Akalux	cetuximab saratolacan	Rakuten Medical	EGFR	IRDye700DX	N/A	r/r head and neck cancer	2020.09
Zynlonta	loncastuximab tesirine	ADC Therapeutics	CD19	PBD	dipeptide	r/r diffuse large B-cell lymphoma	2021.04
Aidixi	disitamab vedotin	Rongchang Biology	HER2	MMAE	mc-VC-PABC	Third-line HER2-positive gastric cancer	2021.06
Tivdak	tisotumab vedotin	Seagen/Genmab	TF	MMAE	mc-VC-PABC	Recurrent or metastatic cervical cancer	2021.09
Elahere	mirvetuximab soravtansine	ImmunoGen	FRa	DM4	sulfo-SPDB	FRat platinum-resistant ovarian cancer	2022.11

### 2.2 Adverse event and identification

Reports involving the fifteen antibody-drug conjugates (ADCs) were identified by a text-string search of each drug by generic name through the FDA public database during a data mining process. We then searched for 15 ADCs one by one, and finally selected eight FDA-approved ADCs. These include gemtuzumab ozogamicin, brentuximab vedotin, trastuzumab emtansine, inotuzumab ozogamicin, polatuzumab vedotin, enfortumab vedotin, trastuzumab deruxtecan, and sacituzumab govitecan. Used as investigational agents to identify AEs associated with cardiovascular disease. Extract AEs markers about these drugs as primary suspects. In openvigil 2.1, information is collected by selecting “ADCs” from the “drug” menu box and setting “role of drug” to “primary suspect.” For cardiovascular disease determination, we conducted a search based on the preferred terms of the Medical Dictionary for drug regulatory activities (MedDRA, version 24.0), through which we selected the categories of cardiovascular events that occurred at a higher rate. It was finally determined that in this study, cardiovascular AEs were divided into 14 narrow SMQs categories as follows:cardiac failure, hypertension, cardiomyopathy, (embolic and thrombotic events, venous), (embolic and thrombotic events, arterial), (embolic and thrombotic events, vessel type unspecified and mixed arterial and venous), haemorrhagic central nervous system vascular conditions, shock-associated circulatory or cardiac conditions (excl torsade de pointes), supraventricular tachyarrhythmias, ischaemic central nervous system vascular conditions, torsade de pointes/QT prolongation, ventricular tachyarrhythmias, other ischaemic heart disease, (cardiac arrhythmia terms, nonspecific) (Table 2). The operation in openvigil 2.1 is as follows: In the adverse event search module, we select “SMQ-narrow” and enter the customized event term “cardiovascular disease” described above. Finally, records were counted according to the Personal Safety Report (ISR). Clinical characteristics of patients were collected independently, including sex, age, report, country and date of receipt of the report, and AEs outcome.

**TABLE 2:** Cardiovascular adverse events grouped into 14 narrow categories of Standardized MedDRA Queries (SMQs) according to MedDRA 24.0.

SMQ name	Algorithm
Cardiac failure	Narrow
Hypertension	Narrow
Cardiomyopathy	Narrow
Embolic and thrombotic events, venous	Narrow
Embolic and thrombotic events, arterial	Narrow
Embolic and thrombotic events, vessel type unspecified and mixed arterial and venous	Narrow
Haemorrhagic central nervous system vascular conditions	Narrow
Shock-associated circulatory or cardiac conditions (excl torsade de pointes)	Narrow
Supraventricular tachyarrhythmias	Narrow
Ischaemic central nervous system vascular conditions	Narrow
Torsade de pointes/QT prolongation	Narrow
Ventricular tachyarrhythmias	Narrow
Other ischaemic heart disease	Narrow
Cardiac arrhythmia terms, nonspecific	Narrow

### 2.3 Statistical analysis

In addition, disproportionation analysis (also known as case-non-case analysis) is a signal detection method based on two-by-two contingency tables (Table 3) that is widely used in pharmacovigilance studies (Ang et al., 2016). It detects potential adverse drug reaction signals by comparing the proportion of target events occurring with the proportion of target events occurring with all other drugs, a table that can be easily created on OpenVigil. Reporting odds ratio (ROR) and proportional reporting ratio of IC are calculated (PRR) to assess the association between adverse events and the drug, their calculation formula (Li et al., 2023), ROR = (a/c)/(b/d), ROR 95% CI = eln ROR ± 1.96 (1/a + 1/b + 1/c + 1/d) 0.5, signal detection threshold: If A ≥ 3 and the lower threshold of 95% CI is greater than 1, an ADR signal is generated. PRR = a/(a + b)]/[c/(c + d)], = (ad-bc) 2 (a + b + c + d)/(a + b) (c + d) (a + c) (b + d)], signal detection threshold: a ≥ 3, PRR≥2and $x^{2}$ ≥ 4, PRR acuity is an ADR signal. The larger the ROR and PRR calculations, the stronger the AE signal, that is, the stronger the statistical correlation between the target drug and the target adverse event (Tang et al., 2022). In this study, when ROR and PRR methods meet the above conditions, they are identified as detected signals indicating an association between drugs and cardiac and cardiovascular events. A descriptive analysis was used to describe the clinical features of the cases. Microsoft Excel 2019 (Microsoft, Redmond, Washington, United States) and SPSS v24.0 (IBM Corp., Armonk) were used for all data management and analysis in this study. (New York, United States). Graphics are illustrated using WPS Office.

**TABLE 3:** 2 × 2 contingency table for disproportionality analysis.

	Drug(s) of interest	All other adverse events	Total
Adverse event(s) of interest	a	b	a + b
All other adverse events	c	d	c + d
Total	a + c	b + d	a + b + c + d

## 3 Results

### 3.1 Descriptive analyses

**FIGURE 1:**
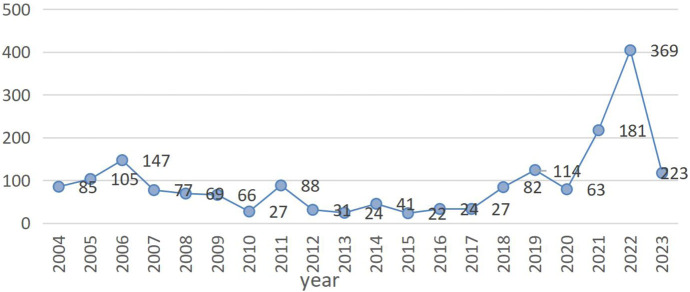
The number of 14 reported cardiovascular adverse events per year after marketing of antibody-drug conjugates (ADCs) in 14 narrow SMQ categories. Note: The blue line represents the eight antibody drug conjugates related to our research. In this figure shows the reported number of cardiovascular events associated with ADCs over time, from 2004 to the second quarter of 2023.

Among them, gemtuzumab ozogamicin reported 779 (41.81%) cases, brentuximab vedotin reported 325 (17.44%) cases, and trastuzumab emtansine reported 101 (5.42%) cases. There were 170 (9.13%) reports related to inotuzumab ozogamicin, 119 (6.39%) reports related to polatuzumab vedotin, 114 (6.12%) reports related to enfortumab vedotin, and 41 (2.2%) reports related to sacituzumab govitecan. Trastuzumab deruxtecan was associated with 214 (11.49%) cases (Supplementary Table S1). The characteristics of 1,863 reports of AEs associated with cardiovascular events submitted for ADCs are shown in (Table 4). Most reports came from people aged ≥65, but a significant number of cases were found to be unknown. The number of patients with antibody-drug conjugates (ADCs)-related CVD cases aged <18 years, 18–64 years, and ≥65 years was 52 (2.79%), 586 (31.45%), and 613 (32.90%), respectively. The proportion of female patients (834, 44.77%) was higher than that of male patients (752, 40.37%). The majority of these reports came from the United States (563, representing 30.22% of the total), followed by 449 (accounting for 24.10%) from Japan. Additionally, 156 reports (constituting 8.38%) were received from France and Canada, while 89 (comprising 4.78%) originated from Germany. Finally, 488 reports (representing 26.19% of the total) came from all other countries. AEs reports peaks in 2022 and 2023, with peaks of 369 and 223, respectively (Figure 1). In addition, adverse reactions leading to hospitalization, whether initial or prolonged, and death were most commonly reported. The characteristics of the AEs report are based on demographics and the severity of the results. In cardiovascular reports with ADCs, men treated with brentuximab vedotin, enfortumab vedotin, polatuzumab vedotin, inotuzumab ozogamicin, and gemtuzumab ozogamicin were more likely to develop CVD than women. However, men treated with trastuzumab emtansine, trastuzumab deruxtecan, and sacituzumab govitecan were less likely to develop CVD than women (Supplementary Table S2).

**TABLE 4:** Reported characteristics associated with eight antibody-drug conjugates (ADCs) from the first quarter of 2004 to the third quarter of 2023.

Characteristics	Antibody-drug conjugate
Number of events	1,863
Age group, *n* (%)	
<18 years	52 (2.79)
18–65 years	586 (31.45)
≥65 years	613 (32.90)
Unknown	612 (32.85)
Patient’s gender, *n* (%)	
Male	752 (40.37)
Female	834 (44.77)
Unknown	277 (14.87)
Reported countries, *n* (%)	
United States	563 (30.22)
Japan	449 (24.10)
France	97 (5.21)
Germany	89 (4.78)
Canada	59 (3.17)
Others countries	488 (26.19)
Unknown	118 (6.33)
Reported year, *n* (%)	
2004	85 (4.56)
2005	105 (5.64)
2006	147 (7.89)
2007	77 (4.13)
2008	69 (3.70)
2009	66 (3.54)
2010	27 (1.45)
2011	88 (4.72)
2012	31 (1.66)
2013	24 (1.29)
2014	41 (2.20)
2015	22 (1.18)
2016	24 (1.29)
2017	27 (1.45)
2018	82 (4.40)
2019	114 (6.12)
2020	63 (3.38)
2021	181 (9.72)
2022	369 (19.81)
2023	223 (11.97)
Outcome of adverse events, *n* (%)	
Death	770 (41.33)
Hospitalization initial or prolonged	407 (21.85)
Life-threatening	187 (10.04)
Disability	9 (0.48)
Required intervention to prevent permanent impairment	8 (0.43)
Other	431 (23.13)
Unknown	51 (2.74)

### 3.2 Cardiovascular AEs profiles of ADCs based on SMQs

Gemtuzumab ozogamicin, brentuximab vedotin, trastuzumab emtansine, inotuzumab ozogamicin, polatuzumab vedotin, enfortumab vedotin, sacituzumab govitecan, and trastuzumab deruxtecan The number of CVD-related SMQS with suspicious signals was 10, 2, 5, 5, 9, 2, 3, and 3 (Supplementary Table S1), respectively. Embolic and thrombotic events, venous (*n* = 446), and cardiac failure (*n* = 277) were the most frequently reported CVD-related AEs in the ADCs class. These PTs differ in their signaling levels in the various ADCs. In terms of longitudinal self-comparison, we examined different signaling levels of cardiovascular disease SMQ across all reported ADC classes of drugs. Gemtuzumab ozogamicin found the most adverse signals among 14 narrow categories of SMQ. Gemtuzumab ozogamicin had the strongest signal for embolic and thrombotic events, venous. ROR and 95% CI, 11.806 (10.259; 13.587). Disproportionate analysis also revealed (Supplementary Table S1) that brentuximab vedotin had the strongest potential association with cardiac failure. ROR and 95% CI, 1.748 (1.351; 2.262) In addition, trastuzumab emtansine had the strongest potential association with cardiomyopathy, ROR and 95% CI, 4.766 (2.759; 8.235). Inotuzumab ozogamicin had the strongest signal for embolic and thrombotic events, venous. ROR and 95% CI, 29.67 (24.242; 36.314). Of the cardiovascular disease-related AEs associated with polatuzumab vedotin, ventricular tachyarrhythmias showed the strongest signal strength (ROR) and 95% CI, 2.302 (0.862; 6.144). Among cardiovascular disease-related AEs associated with enfortumab vedotin, embolic and thrombotic events, venous showed the strongest signal strength of ROR and 95% CI, 1.628 (1.023; 2.591). Among the cardiovascular disease-related adverse events associated with sacituzumab govitecan, ventricular tachyarrhythmias, venous showed the strongest signal strength ROR and 95% CI, 3.013 (0.969; 9.367). Among cardiovascular disease-related AEs associated with trastuzumab deruxtecan, cardiomyopathy showed the strongest signal strength, ROR and 95% CI, 4.433 (3.144; 6.251). Thus, ADCs show a strong signal in embolic and thrombotic events, venous. The other reporting odds ratio (ROR) and proportional reporting ratio (PRR) of the correlation between different drugs and cardiovascular disease (CVD) are shown in (Supplementary Table S1).

### 3.3 Outcome of adverse events

Of note, of all 14 narrow SMQ-class cardiovascular AEs associated with antibody-drug conjugates (ADCs), in 1863 ADC-associated CVD cases (Table 4), the final outcomes were as follows: Death 770 (41.3%)reports, disability 9 (0.5%)reports, Hospitalization initial or prolonged 407 (21.8%)reports, and life-threatening reactions187 (10.0%) reports. Of the 770 (41.3%) deaths reported, 103 (31.7%) were associated with brentuximab vedotin, 10 (24.4%) with sacituzumab govitecan, 22 (19.3%) with enfortumab vedotin, and 35 (34.7%) with trastuzumab emtansine.49 (41.2%) cases were associated with polatuzumab vedotin, 62 (29%) with trastuzumab deruxtecan, 423 (54.3%) with gemtuzumab ozogamicin, and 66 (38.8%) with inotuzumab ozogamicin (Supplementary Table S2). No actual cause-and-effect assessment was performed between ADCs and death, and the data only provided a safety signal, not a real risk. At most 770 (41.3%) deaths. Specifically, the outcomes of higher risk levels, death, life-threatening events, the proportion of hospitalizations corresponding to different signal-significant SMQS, and those resulting in longer hospitalizations. Notably, among the adverse events, hypertension (SMQ) accounts for the lowest proportion in both disability (0.05%) and life-threatening (0.27%) events. However, the adverse events that cause higher mortality are embolic and thrombotic events, venous (SMQ), cardiac failure (SMQ), haemorrhagic central nervous system vascular conditions (SMQ), embolic and thrombotic events, vessel type unspecified and mixed arterial and venous (SMQ), and shock-associated circulatory or cardiac conditions (excl torsade de pointes) (SMQ). The number of deaths caused by these adverse events is 166, 112, 110, 92, and 90, respectively.

## 4 Discussion

With the escalating use of ADCs, an increasing number of adverse reactions related to cardiac toxicity have been reported. The toxic effects of ADCs can be divided into on-target toxicity and off-target toxicity. A typical example of ADC’s on-target toxicity is the cardiac toxicity of anti-HER2 drugs. Cardiotoxicity is a common toxicity of anti-HER2 drugs, which is usually manifested as a decrease in left ventricular ejection fraction (LVEF) and occasionally as congestive heart failure (CHF) (Pondé et al., 2016). The cardiac toxicity of anti-HER2 drugs may be related to the important role of HER2 signaling pathway in myocardial cell function (Pondé et al., 2016). In the EMILIA study, the incidence of LVEF decline >15% or LVEF <50% in the trastuzumab emtansine group was 1.7% (Bregni et al., 2016). Long-term follow-up results showed that the incidence of ≥ grade 3 cardiac insufficiency was <1% (Moilanen et al., 2018; Li et al., 2023). In the KATHERINE study, the incidence of cardiac events in the trastuzumab emtansine group was 0.1% (von Minckwitz et al., 2019). In addition, adverse reactions related to cardiac toxicity have also been reported in previous clinical trials of other ADCs, for instance. The safety and efficacy evaluation of gemtuzumab ozogamicin as monotherapy was performed in the AML-19 and MyloFrance-1 studies. The AEs that occurred were mainly cardiovascular, with a 6% incidence (Amadori et al., 2016). In addition, in a phase 3 trial of inotuzumab ozogamicin (InO), among patients undergoing follow-up HSCT, 53 of 79 (67.1%) in the InO group died of cardiac disease [4 of 29 (13.8%)] (Kantarjian et al., 2019). From the current clinical data, it can be seen that ADCs can lead to cardiovascular adverse reactions, its cardiac toxicity can seriously affect the survival and prognosis of patients. Therefore, it is important for us to raise awareness of its cardiac toxicity. It is also very important for clinicians to rationalize the use of ADCs in clinical practice, strengthen the effective management of adverse reactions to cardiac toxicity, and maximize the clinical effectiveness of the drugs, which also affects the survival and outcome of patients.

Currently, there is a lack of real-world data, and ADCs are gradually being launched in China and widely used in clinical practice. Therefore, we need to conduct signal mining and analysis based on databases to explore the cardiac safety issues after their launch, providing a reference for safe and rational clinical use of drugs. This study explores the risk status of ADCs from the perspective of signal risk by accessing cardiovascular AEs data from the FAERS database and conducting signal mining on the FAERS database. The results of this study showed that cardiovascular AEs were collected in 8 out of 15 ADCs. The reason for zero reports registering belantamab madotin (approved in 2020), tisatumab vedotin (approved in 2021), cetuximab saratolacan (approved in 2020), disitamab vedotin (approved in 2021), mirvetuximab soravtansine (approved in 2022), and loncastuximab tesirine (approved in 2021) is mainly due to the short time they have been on the market. The reason for zero reports reporting on moxetumab pasudotox may be related to the small number of patients who received treatment after the drug was marketed. Among the 1,863 disease lines collected from 8 ADCs, gemtuzumab ozogamycin had the highest number of cases, and gemtuzumab ozogamycin was strongly associated with CVD according to the PRR and ROR methods. PRR and ROR methods, The sacituzumab-govitecan signal is weak. This may be related to the short launch time, with gemtuzumab ozogamicin being the earliest to be launched. Sacituzumab govitecan was only approved for listing in 2020. However, the signals we observed indicate that the strength of signals corresponding to different cardiovascular types varies among different drugs. For example, gemtuzumab ozogamicin exhibits the strongest signal for embolic and thrombotic events, venous, while brentuximab vedotin demonstrates the strongest signal for cardiac failure. This suggests that the manifestations of cardiovascular toxicity vary among different drugs. According to the year-end analysis, the report reached its peak in 2022. The reason may be that over time, more and more ADCs have been launched, and in 2023 (Figure 1), there has been a decline. In fact, this is because only two-quarters were collected in 2023. Further research is needed to verify the actual needs. Most ADCs have been listed since 2011. Gemtuzumab ozogamicin (Wyeth/Pfizer) received accelerated approval for relapsed CD33^+^ acute myeloid leukemia (AML) in 2000 but was voluntarily withdrawn from the market in 2010 after post-marketing studies. In 2017, the FDA re-approved alternative doses of the drug.

Through gender and age analysis, the proportion of cardiovascular disease in women is higher than that in men; patients over 65 years old are more prone to cardiovascular AEs. The higher proportion of female patients may be related to the indications of ADC drugs. Trastuzumab deruxtecan and trastuzumab emtansine are used to treat breast cancer patients, which may be the reason for the higher number of female patients than male patients. Although the proportion of female patients is higher than male patients, it is currently unclear whether the actual proportion of men and women using these ADC drugs is balanced. The reason for the high proportion of patients over 65 years old may be that patients under 65 years old have better tolerance. Although the number of cardiovascular events caused by ADCs together is higher in patients over 65 years old and more in women than men, there are differences between different drugs. As far as the country is concerned, the United States has the highest number of reported cases. This may be related to the fact that most ADCs have been approved for marketing in the United States. In terms of outcomes, of the 1863 patients who developed ADC-related cardiovascular events, 770 (41.33%) died during treatment. The correlation between this death outcome and ADCs needs further research.

Therefore, our research indicates that there is a certain signal link between these eight ADCs and cardiovascular issues, which proves that these eight ADC drugs pose a cardiovascular risk. Maintaining a healthy cardiovascular system is essential for the survival and wellbeing of mammalian life. Because the development of cardiovascular disease (CVD) is a major determinant of human morbidity and mortality, cardiovascular disease (CVD) has become the leading cause of non-relapse-related death among cancer survivors (Wong-Siegel et al., 2023). Cardiovascular toxicity (CT) includes but is not limited to hypertension, arrhythmia, and cardiomyopathy, with 1,000 definitions of cardiovascular relevance initially screened in the International ICD10 Diagnostic Code. In our study, we also provided additional information on the association between ADC use and cardiovascular-associated AEs. Mortality from CVD and other causes is clinically significant in the long-term follow-up of cancer patients (Clèries et al., 2022). Because cardiovascular disease severity is common in patients, further research into ADC cardiovascular disease is of clear importance for human health. Our research is based on the FAERS database, but there are also some limitations in our study. These limitations include the following points. 1) Antibody-drug conjugates (ADCs) have been on the market for a short time worldwide, and ADR monitoring data are relatively incomplete. 2) The hypothesis generated by the pharmacovigilance database only observed signals, but it cannot verify the signal detection related to the hypothesis, and the relevant detection needs to be verified through prospective studies. 3) As the FAERS database is a self-reporting system, there may be omissions, biases, and inconsistencies in the vast amount of information, and the resulting biases may affect the outcome of data mining. Similar to all drug safety studies, this database does not provide diagnostic information, therefore, it is not possible to analyze and prove the relationship between adverse reactions and drugs. 4) Currently, ADCs are gradually being launched in China. Due to the short period of time since their launch and the limited types available, there is a lack of real-world data. However, as the use of these drugs increases domestically in the future, we can utilize domestic real-world data for further exploration. 5) The analysis based on the existing information in the FAERS database is not clear about whether patients have cardiovascular disease at baseline, which may affect the analysis of the results.

Although this study identified many adverse effects not mentioned in the description of the ADCs, causality needs to be further evaluated. Future studies can be considered.

## 5 Conclusion

The FAERS data mining has shown that cardiovascular AEs are associated with Ado-gemtuzumab ozogamicin, brentuximab vedotin, trastuzumab emtansine, inotuzumab ozogamicin, polatuzumab vedotin, enfortumab vedotin, trastuzumab deruxtecan, and sacituzumab govitecan. ADCs have not been analyzed in a large post-marketing population. Based on the FAERS database, the ADRs of ADCs can be deeply detected from various aspects, which can be used as a supplement to the drug label or specification. We recommend that clinicians regularly monitor ADCs and be alert for adverse reactions associated with cardiovascular disease in patients receiving ADCs. The safety of ADCs must be continually explored in the real world to better protect patients.

## Data Availability

The raw data supporting the conclusions of this article will be made available by the authors, without undue reservation.
