# Supplementary material for: Cardiovascular adverse events associated with antibody-drug conjugates (ADCs): a pharmacovigilance study based on the FAERS database
Source: Front Pharmacol. 2024 May 3;15:1378010. doi: 10.3389/fphar.2024.1378010 (PMC11099241; doi:10.3389/fphar.2024.1378010)
Supplement: Supplementary file 1 [file Table1.DOCX]

**Supplement table 1. Counts of Cardiovascular disease（CVD） with associated RORs and PRRs for ADCs from the FAERS database.**

| gemtuzumab ozogamicin | | | | |
| --- | --- | --- | --- | --- |
| SMQ | N | (PRR) and 95% CI | (ROR) and 95% CI | X2 |
| cardiac failure | 110 | 4.598 ( 3.834 ; 5.515 ) | 4.808 ( 3.967 ; 5.828 ) | 309.645 |
| hypertension | 38 | 1.028 ( 0.75 ; 1.409 ) | 1.029 ( 0.746 ; 1.418 ) | 0.008 |
| cardiomyopathy | 29 | 5.246 ( 3.655 ; 7.53 ) | 5.309 ( 3.679 ; 7.661 ) | 95.647 |
| embolic and thrombotic events, venous | 219 | 10.62 ( 9.371 ; 12.035 ) | 11.806 ( 10.259 ; 13.587 ) | 1915.515 |
| embolic and thrombotic events, arterial | 51 | 1.315 ( 1.003 ; 1.724 ) | 1.323 ( 1.002 ; 1.747 ) | 3.609 |
| embolic and thrombotic events, vessel type unspecified and mixed arterial and venous | 74 | 1.751 ( 1.401 ; 2.19 ) | 1.78 ( 1.411 ; 2.246 ) | 23.607 |
| haemorrhagic central nervous system vascular conditions | 81 | 2.775 ( 2.242 ; 3.435 ) | 2.85 ( 2.282 ; 3.56 ) | 91.514 |
| shock-associated circulatory or cardiac conditions (excl torsade de pointes) | 65 | 3.438 ( 2.706 ; 4.366 ) | 3.52 ( 2.749 ; 4.507 ) | 110.92 |
| supraventricular tachyarrhythmias | 46 | 4.046 ( 3.041 ; 5.385 ) | 4.118 ( 3.074 ; 5.518 ) | 103.006 |
| ischaemic central nervous system vascular conditions | 19 | 0.621 ( 0.397 ; 0.972 ) | 0.617 ( 0.393 ; 0.97 ) | 4.084 |
| cardiac arrhythmia terms, nonspecific | 9 | 1.222 ( 0.637 ; 2.346 ) | 1.223 ( 0.635 ; 2.354 ) | 0.176 |
| torsade de pointes/QT prolongation | 13 | 2.376 ( 1.382 ; 4.085 ) | 2.385 ( 1.382 ; 4.115 ) | 9.05 |
| ventricular tachyarrhythmias | 12 | 2.778 ( 1.58 ; 4.884 ) | 2.789 ( 1.581 ; 4.919 ) | 11.955 |
| other ischaemic heart disease | 13 | 1.163 ( 0.677 ; 2.0 ) | 1.165 ( 0.675 ; 2.009 ) | 0.158 |
| brentuximab vedotin | | | | |
| SMQ |  | (PRR) and 95% CI | (ROR) and 95% CI | X2 |
| cardiac failure | 59 | 1.735 ( 1.347 ; 2.234) | 1.748 ( 1.351 ; 2.262 ) | 17.814 |
| hypertension | 11 | 0.199 ( 0.11 ; 0.359 ) | 0.196 ( 0.109 ; 0.355 ) | 35.24 |
| cardiomyopathy | 10 | 1.23 ( 0.662 ; 2.284 ) | 1.231 ( 0.661 ; 2.289 ) | 0.231 |
| embolic and thrombotic events, venous | 38 | 1.375 ( 1.002 ; 1.886 ) | 1.379 ( 1.001 ; 1.899 ) | 3.541 |
| embolic and thrombotic events, arterial | 28 | 0.62 ( 0.428 ; 0.896 ) | 0.616 ( 0.425 ; 0.894 ) | 6.251 |
| embolic and thrombotic events, vessel type unspecified and mixed arterial and venous | 55 | 0.972 ( 0.748 ; 1.263 ) | 0.971 ( 0.744 ; 1.268 ) | 0.021 |
| haemorrhagic central nervous system vascular conditions | 24 | 0.622 ( 0.417 ; 0.926 ) | 0.619 ( 0.414 ; 0.925 ) | 5.212 |
| shock-associated circulatory or cardiac conditions (excl torsade de pointes) | 31 | 1.209 ( 0.852 ; 1.717 ) | 1.211 ( 0.85 ; 1.726 ) | 0.931 |
| supraventricular tachyarrhythmias | 23 | 1.374 ( 0.914 ; 2.065 ) | 1.377 ( 0.913 ; 2.075 ) | 1.992 |
| ischaemic central nervous system vascular conditions | 24 | 0.618 ( 0.415 ; 0.92 ) | 0.615 ( 0.412 ; 0.919 ) | 5.363 |
| cardiac arrhythmia terms, nonspecific | 5 | 0.507 ( 0.211 ; 1.217 ) | 0.506 ( 0.211 ; 1.217 ) | 1.936 |
| torsade de pointes/QT prolongation | 3 | 0.361 ( 0.116 ; 1.119 ) | 0.36 ( 0.116 ; 1.118 ) | 2.79 |
| ventricular tachyarrhythmias | 3 | 0.534 ( 0.172 ; 1.656 ) | 0.534 ( 0.172 ; 1.656 ) | 0.798 |
| other ischaemic heart disease | 11 | 0.858 ( 0.476 ; 1.548 ) | 0.858 ( 0.474 ; 1.55 ) | 0.136 |
| trastuzumab emtansine | | | | |
| SMQ |  | (PRR) and 95% CI | (ROR) and 95% CI | X2 |
| cardiac failure | 17 | 1.524 ( 0.951 ; 2.443 ) | 1.532 ( 0.949 ; 2.473 ) | 2.585 |
| hypertension | 5 | 0.267 ( 0.111 ; 0.641 ) | 0.264 ( 0.11 ; 0.636 ) | 9.481 |
| cardiomyopathy | 13 | 4.724 ( 2.751 ; 8.112 ) | 4.766 ( 2.759 ; 8.235 ) | 34.593 |
| embolic and thrombotic events, venous | 6 | 0.714 ( 0.322 ; 1.587 ) | 0.713 ( 0.32 ; 1.59 ) | 0.433 |
| embolic and thrombotic events, arterial | 2 | 0.139 ( 0.035 ; 0.555 ) | 0.137 ( 0.034 ; 0.55 ) | 9.961 |
| embolic and thrombotic events, vessel type unspecified and mixed arterial and venous | 14 | 0.748 ( 0.441 ; 1.266 ) | 0.748 ( 0.441 ; 1.266 ) | 0.939 |
| haemorrhagic central nervous system vascular conditions | 17 | 1.332 ( 0.831 ; 2.135 ) | 1.337 ( 0.828 ; 2.158 ) | 1.106 |
| shock-associated circulatory or cardiac conditions (excl torsade de pointes) | 10 | 1.206 ( 0.651 ; 2.236 ) | 1.208 ( 0.648 ; 2.251 ) | 0.177 |
| supraventricular tachyarrhythmias | 1 | 0.178 ( 0.025 ; 1.26 ) | 0.177 ( 0.025 ; 1.257 ) | 3.042 |
| ischaemic central nervous system vascular conditions | 7 | 0.555 ( 0.265 ; 1.161 ) | 0.552 ( 0.263 ; 1.16 ) | 2.1 |
| cardiac arrhythmia terms, nonspecific | 2 | 0.625 ( 0.156 ; 2.494 ) | 0.624 ( 0.156 ; 2.498 ) | 0.155 |
| torsade de pointes/QT prolongation | 2 | 0.699 ( 0.175 ; 2.792 ) | 0.699 ( 0.174 ; 2.797 ) | 0.046 |
| ventricular tachyarrhythmias | 3 | 1.623 ( 0.524 ; 5.024 ) | 1.624 ( 0.523 ; 5.044 ) | 0.23 |
| other ischaemic heart disease | 2 | 0.499 ( 0.125 ; 1.994 ) | 0.498 ( 0.124 ; 1.995 ) | 0.568 |
| inotuzumab ozogamicin | | | | |
| SMQ |  | (PRR) and 95% CI | (ROR) and 95% CI | X2 |
| cardiac failure | 7 | 1.128 ( 0.54 ; 2.358 ) | 1.13 ( 0.536 ; 2.378 ) | 0.014 |
| hypertension | 3 | 0.248 ( 0.08 ; 0.768 ) | 0.245 ( 0.079 ; 0.761 ) | 6.208 |
| cardiomyopathy | 1 | 0.562 ( 0.079 ; 3.988 ) | 0.562 ( 0.079 ; 3.994 ) | 0.044 |
| embolic and thrombotic events, venous | 112 | 25.135 ( 21.202 ; 29.797 ) | 29.67 ( 24.242 ; 36.314 ) | 2581.326 |
| embolic and thrombotic events, arterial | 8 | 1.163 ( 0.584 ; 2.316 ) | 1.165 ( 0.58 ; 2.338 ) | 0.056 |
| embolic and thrombotic events, vessel type unspecified and mixed arterial and venous | 11 | 1.041 ( 0.579 ; 1.871 ) | 1.041 ( 0.574 ; 1.889 ) | 0 |
| haemorrhagic central nervous system vascular conditions | 19 | 2.776 ( 1.781 ; 4.326 ) | 2.825 ( 1.791 ; 4.457 ) | 20.038 |
| shock-associated circulatory or cardiac conditions (excl torsade de pointes) | 0 | / | / | / |
| supraventricular tachyarrhythmias | 0 | / | / | / |
| ischaemic central nervous system vascular conditions | 6 | 0.877 ( 0.395 ; 1.946 ) | 0.876 ( 0.392 ; 1.957 ) | 0.017 |
| cardiac arrhythmia terms, nonspecific | 1 | 0.497 ( 0.07 ; 3.526 ) | 0.497 ( 0.07 ; 3.53 ) | 0.13 |
| torsade de pointes/QT prolongation | 0 | / | / | / |
| ventricular tachyarrhythmias | 2 | 1.852 ( 0.464 ; 7.393 ) | 1.855 ( 0.463 ; 7.431 ) | 0.164 |
| other ischaemic heart disease | 0 | / | / | / |
| polatuzumab vedotin | | | | |
| SMQ |  | (PRR) and 95% CI | (ROR) and 95% CI | X2 |
| cardiac failure | 23 | 2.169 ( 1.447 ; 3.252 ) | 2.191 ( 1.451 ; 3.311 ) | 13.459 |
| hypertension | 2 | 0.092 ( 0.023 ; 0.366 ) | 0.09 ( 0.023 ; 0.361 ) | 17.41 |
| cardiomyopathy | 7 | 2.19 ( 1.046 ; 4.586 ) | 2.197 ( 1.045 ; 4.62 ) | 3.423 |
| embolic and thrombotic events, venous | 11 | 1.46 ( 0.811 ; 2.63 ) | 1.464 ( 0.809 ; 2.652 ) | 1.176 |
| embolic and thrombotic events, arterial | 3 | 0.26 ( 0.084 ; 0.805 ) | 0.258 ( 0.083 ; 0.801 ) | 5.656 |
| embolic and thrombotic events, vessel type unspecified and mixed arterial and venous | 17 | 0.921 ( 0.575 ; 1.477 ) | 0.92 ( 0.57 ; 1.485 ) | 0.05 |
| haemorrhagic central nervous system vascular conditions | 14 | 1.255 ( 0.745 ; 2.113 ) | 1.258 ( 0.743 ; 2.13 ) | 0.496 |
| shock-associated circulatory or cardiac conditions (excl torsade de pointes) | 8 | 1.023 ( 0.513 ; 2.041 ) | 1.023 ( 0.51 ; 2.05 ) | 0.013 |
| supraventricular tachyarrhythmias | 9 | 1.42 ( 0.74 ; 2.722 ) | 1.423 ( 0.738 ; 2.741 ) | 0.74 |
| ischaemic central nervous system vascular conditions | 8 | 0.697 ( 0.349 ; 1.39 ) | 0.695 ( 0.347 ; 1.393 ) | 0.781 |
| cardiac arrhythmia terms, nonspecific | 7 | 2.035 ( 0.972 ; 4.261 ) | 2.041 ( 0.971 ; 4.291 ) | 2.729 |
| torsade de pointes/QT prolongation | 3 | 1.043 ( 0.337 ; 3.229 ) | 1.043 ( 0.336 ; 3.239 ) | 0.05 |
| ventricular tachyarrhythmias | 4 | 2.298 ( 0.863 ; 6.114 ) | 2.302 ( 0.862 ; 6.144 ) | 1.778 |
| other ischaemic heart disease | 3 | 0.89 ( 0.287 ; 2.757 ) | 0.89 ( 0.287 ; 2.764 ) | 0.005 |
| enfortumab vedotin | | | | |
| SMQ |  | (PRR) and 95% CI | (ROR) and 95% CI | X2 |
| cardiac failure | 12 | 0.77 ( 0.438 ; 1.353 ) | 0.768 ( 0.435 ; 1.355 ) | 0.618 |
| hypertension | 3 | 0.092 ( 0.03 ; 0.285 ) | 0.09 ( 0.029 ; 0.281 ) | 26.469 |
| cardiomyopathy | 0 | / | / | / |
| embolic and thrombotic events, venous | 18 | 1.622 ( 1.024 ; 2.569 ) | 1.628 ( 1.023 ; 2.591 ) | 3.716 |
| embolic and thrombotic events, arterial | 13 | 0.767 ( 0.446 ; 1.319 ) | 0.766 ( 0.444 ; 1.321 ) | 0.707 |
| embolic and thrombotic events, vessel type unspecified and mixed arterial and venous | 27 | 0.982 ( 0.675 ; 1.428 ) | 0.982 ( 0.671 ; 1.436 ) | 0 |
| haemorrhagic central nervous system vascular conditions | 6 | 0.367 ( 0.165 ; 0.815 ) | 0.364 ( 0.164 ; 0.812 ) | 6.002 |
| shock-associated circulatory or cardiac conditions (excl torsade de pointes) | 4 | 0.349 ( 0.131 ; 0.929 ) | 0.348 ( 0.13 ; 0.928 ) | 4.25 |
| supraventricular tachyarrhythmias | 12 | 1.269 ( 0.722 ; 2.23 ) | 1.27 ( 0.72 ; 2.241 ) | 0.442 |
| ischaemic central nervous system vascular conditions | 9 | 0.53 ( 0.276 ; 1.016 ) | 0.527 ( 0.274 ; 1.015 ) | 3.332 |
| cardiac arrhythmia terms, nonspecific | 4 | 0.79 ( 0.297 ; 2.104 ) | 0.79 ( 0.296 ; 2.107 ) | 0.062 |
| torsade de pointes/QT prolongation | 1 | 0.24 ( 0.034 ; 1.705 ) | 0.24 ( 0.034 ; 1.704 ) | 1.705 |
| ventricular tachyarrhythmias | 1 | 0.398 ( 0.056 ; 2.822 ) | 0.397 ( 0.056 ; 2.823 ) | 0.41 |
| other ischaemic heart disease | 4 | 0.797 ( 0.299 ; 2.122 ) | 0.797 ( 0.299 ; 2.125 ) | 0.054 |
| sacituzumab govitecan | | | | |
| SMQ |  | (PRR) and 95% CI | (ROR) and 95% CI | X2 |
| cardiac failure | 1 | 0.161 ( 0.023 ; 1.145 ) | 0.16 ( 0.023 ; 1.14 ) | 3.587 |
| hypertension | 1 | 0.077 ( 0.011 ; 0.543 ) | 0.075 ( 0.011 ; 0.535 ) | 10.414 |
| cardiomyopathy | 0 | / | / | / |
| embolic and thrombotic events, venous | 9 | 2.045 ( 1.068 ; 3.915 ) | 2.058 ( 1.066 ; 3.972 ) | 3.84 |
| embolic and thrombotic events, arterial | 1 | 0.149 ( 0.021 ; 1.055 ) | 0.148 ( 0.021 ; 1.049 ) | 4.094 |
| embolic and thrombotic events, vessel type unspecified and mixed arterial and venous | 8 | 0.732 ( 0.368 ; 1.459 ) | 0.729 ( 0.363 ; 1.464 ) | 0.546 |
| haemorrhagic central nervous system vascular conditions | 5 | 0.779 ( 0.325 ; 1.866 ) | 0.778 ( 0.323 ; 1.874 ) | 0.132 |
| shock-associated circulatory or cardiac conditions (excl torsade de pointes) | 7 | 1.543 ( 0.738 ; 3.227 ) | 1.549 ( 0.736 ; 3.26 ) | 0.856 |
| supraventricular tachyarrhythmias | 1 | 0.264 ( 0.037 ; 1.873 ) | 0.263 ( 0.037 ; 1.871 ) | 1.386 |
| ischaemic central nervous system vascular conditions | 4 | 0.595 ( 0.224 ; 1.582 ) | 0.593 ( 0.222 ; 1.585 ) | 0.739 |
| cardiac arrhythmia terms, nonspecific | 0 | / | / | / |
| torsade de pointes/QT prolongation | 1 | 0.617 ( 0.087 ; 4.375 ) | 0.617 ( 0.087 ; 4.383 ) | 0.009 |
| ventricular tachyarrhythmias | 3 | 3.005 ( 0.971 ; 9.299 ) | 3.013 ( 0.969 ; 9.367 ) | 2.26 |
| other ischaemic heart disease | 0 | / | / | / |
| trastuzumab deruxtecan | | | | |
| SMQ |  | (PRR) and 95% CI | (ROR) and 95% CI | X2 |
| cardiac failure | 48 | 1.967 ( 1.485 ; 2.604 ) | 1.983 ( 1.491 ; 2.638 ) | 22.016 |
| hypertension | 9 | 0.176 ( 0.092 ; 0.338 ) | 0.174 ( 0.09 ; 0.334 ) | 34.478 |
| cardiomyopathy | 33 | 4.394 ( 3.129 ; 6.172 ) | 4.433 ( 3.144 ; 6.251 ) | 83.138 |
| embolic and thrombotic events, venous | 33 | 1.9 ( 1.353 ; 2.667 ) | 1.91 ( 1.355 ; 2.692 ) | 13.237 |
| embolic and thrombotic events, arterial | 9 | 0.339 ( 0.177 ; 0.651 ) | 0.337 ( 0.175 ; 0.648 ) | 11.045 |
| embolic and thrombotic events, vessel type unspecified and mixed arterial and venous | 31 | 0.72 ( 0.507 ; 1.022 ) | 0.717 ( 0.503 ; 1.021 ) | 3.148 |
| haemorrhagic central nervous system vascular conditions | 24 | 0.936 ( 0.629 ; 1.395 ) | 0.936 ( 0.626 ; 1.399 ) | 0.05 |
| shock-associated circulatory or cardiac conditions (excl torsade de pointes) | 4 | 0.223 ( 0.084 ; 0.593 ) | 0.222 ( 0.083 ; 0.591 ) | 10.136 |
| supraventricular tachyarrhythmias | 7 | 0.472 ( 0.225 ; 0.99 ) | 0.471 ( 0.224 ; 0.989 ) | 3.634 |
| ischaemic central nervous system vascular conditions | 16 | 0.601 ( 0.369 ; 0.98 ) | 0.599 ( 0.366 ; 0.979 ) | 3.876 |
| cardiac arrhythmia terms, nonspecific | 0 | / | / | / |
| torsade de pointes/QT prolongation | 0 | / | / | / |
| ventricular tachyarrhythmias | 0 | / | / | / |
| other ischaemic heart disease | 0 | / | / | / |
