# Supplementary material for: Cardiovascular adverse events associated with antibody-drug conjugates (ADCs): a pharmacovigilance study based on the FAERS database
Source: Front Pharmacol. 2024 May 3;15:1378010. doi: 10.3389/fphar.2024.1378010 (PMC11099241; doi:10.3389/fphar.2024.1378010)
Supplement: Supplementary file 2 [file Table2.DOCX]

**Supplement table 2.The characteristics of cardiovascular adverse reactions are associated with ADCs.**

| Characteristics | Sacituzumab govitecan(n，%) | Enfortumab vedotin(n，%) | Brentuximab vedotin(n，%) | Trastuzumab emtansine(n，%) | Polatuzumab vedotin(n，%) | Inotuzumab ozogamicin(n，%) | Gemtuzumab ozogamicin(n，%) | Trastuzumab deruxtecan(n，%) |
| --- | --- | --- | --- | --- | --- | --- | --- | --- |
| Age group |  |  |  |  |  |  |  |  |
| Unknown | 22（53.7） | 13（11.4） | 130（40.0） | 43（42.6） | 16（13.4） | 57（33.5） | 229（29.4） | 102（47.7） |
| <18 | 0（0） | 0（0） | 8（2.5） | 0（0） | 0（0） | 18（10.6） | 26（3.3） | 0（0） |
| >=65 | 10（24.4） | 82（71.9） | 90（27.7） | 13（12.9） | 85（71.4） | 20（11.8） | 252（32.3） | 61（28.5） |
| 18-65 | 9（22.0） | 19（16.7） | 97（29.8） | 45（44.6） | 18（15.1） | 75（44.1） | 272（34.9） | 51（23.8） |
| Gender |  |  |  |  |  |  |  |  |
| F | 38（92.7） | 18（15.8） | 84（25.8） | 89（88.1） | 50（42.0） | 51（30.0） | 355（45.6） | 149（69.6） |
| M | 3（7.3） | 91（79.8） | 136（41.8） | 1（1.0） | 53（44.5） | 73（42.9） | 358（46.0） | 37（17.3） |
| Unknown | 0（0） | 5（4.4） | 105（32.3） | 11（10.9） | 16（13.4） | 46（27.1） | 66（8.5） | 28（13.1） |
| Countries |  |  |  |  |  |  |  |  |
| United States | 10（24.4） | 26（22.8） | 94（28.9） | 15（14.9） | 3（2.5） | 65（38.2） | 284（36.5） | 66（30.8） |
| Japan | 0（0） | 69（60.5） | 77（23.7） | 18（17.8） | 77（64.7） | 47（27.6） | 97（12.5） | 64（29.9） |
| France | 8（19.5） | 3（2.6） | 25（7.7） | 5（5.0） | 0（0） | 10（5.9） | 39（5.0） | 7（3.3） |
| Germany | 1（2.4） | 1（0.9） | 42（12.9） | 3（3.0） | 1（0.8） | 6（3.5） | 26（3.3） | 9（4.2） |
| Canada | 3（7.3） | 0（0） | 1（0.3） | 3（3.0） | 0（0） | 6（3.5） | 3（0.4） | 43（20.1） |
| Others countries | 19（46.3） | 15（13.2） | 86（26.5） | 57（56.4） | 38（31.9） | 36（21.2） | 212（27.2） | 25（11.7） |
| Unknown | 0（0） | 0（0） | 0（0） | 0（0） | 0（0） | 0（0） | 118（15.1） | 0（0） |
| Date received |  |  |  |  |  |  |  |  |
| 2004 | 0（0） | 0（0） | 0（0） | 0（0） | 0（0） | 0（0） | 85（10.9） | 0（0） |
| 2005 | 0（0） | 0（0） | 0（0） | 0（0） | 0（0） | 0（0） | 103（13.2） | 0（0） |
| 2006 | 0（0） | 0（0） | 0（0） | 0（0） | 0（0） | 0（0） | 147（18.9） | 0（0） |
| 2007 | 0（0） | 0（0） | 0（0） | 0（0） | 0（0） | 0（0） | 77（9.9） | 0（0） |
| 2008 | 0（0） | 0（0） | 0（0） | 0（0） | 0（0） | 0（0） | 69（8.9） | 0（0） |
| 2009 | 0（0） | 0（0） | 0（0） | 0（0） | 0（0） | 0（0） | 66（8.5） | 0（0） |
| 2010 | 0（0） | 0（0） | 0（0） | 0（0） | 0（0） | 0（0） | 27（3.5） | 0（0） |
| 2011 | 0（0） | 0（0） | 5（1.5） | 0（0） | 0（0） | 0（0） | 83（10.7） | 0（0） |
| 2012 | 0（0） | 0（0） | 25（7.7） | 0（0） | 0（0） | 0（0） | 6（0.8） | 0（0） |
| 2013 | 0（0） | 0（0） | 15（4.6） | 6（5.9） | 0（0） | 0（0） | 3（0.4） | 0（0） |
| 2014 | 0（0） | 0（0） | 29（8.9） | 11（10.9） | 0（0） | 0（0） | 1（0.1） | 0（0） |
| 2015 | 0（0） | 0（0） | 18（5.5） | 1（1.0） | 0（0） | 0（0） | 3（0.4） | 0（0） |
| 2016 | 0（0） | 0（0） | 24（7.4） | 0（0） | 0（0） | 0（0） | 0（0） | 0（0） |
| 2017 | 0（0） | 0（0） | 20（6.2） | 4（4.0） | 0（0） | 1（0.6） | 2（0.3） | 0（0） |
| 2018 | 0（0） | 0（0） | 39（12.0） | 1（1.0） | 0（0） | 29（17.1） | 13（1.7） | 0（0） |
| 2019 | 0（0） | 0（0） | 43（13.2） | 2（2.0） | 2（1.7） | 37（21.8） | 30（3.9） | 0（0） |
| 2020 | 0（0） | 3（2.6） | 18（5.5） | 13（12.9） | 6（5.0） | 14（8.2） | 6（0.8） | 3（1.4） |
| 2021 | 2（4.9） | 10（8.8） | 32（9.8） | 19（18.8） | 22（18.5） | 39（22.9） | 25（3.2） | 32（15.0） |
| 2022 | 30（73.2） | 65（57.0） | 42（12.9） | 29（28.7） | 62（52.1） | 30（17.6） | 18（2.3） | 93（43.5） |
| 2023 | 9（22.0） | 36（31.6） | 15（4.6） | 15（14.9） | 27（22.7） | 20（11.8） | 15（1.9） | 86（40.2） |
| Outcome |  |  |  |  |  |  |  |  |
| Death | 10（24.4） | 22（19.3） | 103（31.7） | 35（34.7） | 49（41.2） | 66（38.8） | 423（54.3） | 62（29.0） |
| Hospitalization initial or prolonged | 20（48.8） | 41（36.0） | 121（37.2） | 20（19.8） | 19（16.0） | 27（15.9） | 117（15.0） | 42（19.6） |
| Life-threatening | 1（2.4） | 3（2.6） | 23（7.1） | 4（4.0） | 2（1.7） | 11（6.5） | 130（16.7） | 13（6.1） |
| Disability | 0（0） | 0（0） | 2（0.6） | 0（0） | 2（1.7） | 0（0） | 3（0.4） | 2（0.9） |
| Required Intervention to Prevent Permanent Impairment | 0（0） | 0（0） | 8（2.5） | 0（0） | 0（0） | 0（0） | 0（0） | 0（0） |
| Other | 10（24.4） | 47（41.2） | 57（17.5） | 42（41.6） | 47（39.5） | 64（37.6） | 100（12.8） | 64（29.9） |
